# The Effect of Dietary Rye Inclusion and Xylanase Supplementation on Structural Organization of Bone Constitutive Phases in Laying Hens Fed a Wheat-Corn Diet

**DOI:** 10.3390/ani10112010

**Published:** 2020-10-31

**Authors:** Siemowit Muszyński, Marta Arczewska, Sylwester Świątkiewicz, Anna Arczewska-Włosek, Piotr Dobrowolski, Izabela Świetlicka, Monika Hułas-Stasiak, Tomasz Blicharski, Janine Donaldson, Tomasz Schwarz, Ewa Tomaszewska

**Affiliations:** 1 Department of Biophysics, Faculty of Environmental Biology, University of Life Sciences in Lublin, 13 Akademicka St., 20-950 Lublin, Poland; marta.arczewska@up.lublin.pl (M.A.); izabela.swietlicka@up.lublin.pl (I.Ś.); 2 Department of Animal Nutrition and Feed Science, National Research Institute of Animal Production, 1 Krakowska St., 32-083 Balice, Poland; sylwester.swiatkiewicz@izoo.krakow.pl (S.Ś.); anna.arczewska@izoo.krakow.pl (A.A.-W.); 3 Department of Functional Anatomy and Cytobiology, Faculty of Biology and Biotechnology, Maria Curie-Sklodowska University, 19 Akademicka St., 20-033 Lublin, Poland; piotr.dobrowolski@umcs.lublin.pl (P.D.); monhul@o2.pl (M.H.-S.); 4 Chair and Department of Rehabilitation and Orthopaedics, Medical University in Lublin, 8 Jaczewskiego St., 20-090 Lublin, Poland; blicharski@vp.pl; 5 School of Physiology, Faculty of Health Sciences, University of the Witwatersrand, 7 York Road, Parktown, Johannesburg 2193, South Africa; janine.donaldson@wits.ac.za; 6 Department of Animal Genetics, Breeding and Ethology, Faculty of Animal Sciences, University of Agriculture in Kraków, 24/28 Mickiewicza Ave., 30-059 Cracow, Poland; rzschwar@cyf-kr.edu.pl; 7 Department of Animal Physiology, Faculty of Veterinary Medicine, University of Life Sciences in Lublin, 12 Akademicka St., 20-950 Lublin, Poland; ewaRST@interia.pl

**Keywords:** laying hen, tibia, rye, xylanase, bone quality, collagen

## Abstract

**Simple Summary:**

Consumption of diets with a high-level incorporation of rye leads to an increase in the concentration of nonstarch polysaccharides, which cannot be utilized by poultry. The enzyme xylanase degrades arabinoxylans, the most common nonstarch polysaccharides present in both wheat and rye. In this study, the effect of dietary hybrid rye inclusion and enzyme xylanase supplementation to a diet of laying hens on bone quality was evaluated. Bone quality in laying hens is especially important as one of the bone functions is to store calcium and other minerals for eggshell production. The results of our study showed that modern hybrid rye varieties, when supplemented with exogenous xylanase, can be introduced to the diet of laying hens without any adverse effects on bone structure.

**Abstract:**

This study was conducted to examine the effect of dietary rye inclusion and xylanase supplementation on the bone quality of ISA Brown laying hens. Ninety-six laying hens were assigned to four groups: fed with wheat–corn diet or rye–wheat–corn diet (25% of hybrid rye inclusion) or nonsupplemented or supplemented with xylanase (200 mg/kg of feed) for a period of 25 weeks, from the 26th to the 50th week of age. X-ray absorptiometry, X-ray diffraction, and Fourier-transform infrared spectroscopy were used to provide comprehensive information about the structural organization of bone constitutive phases of the tibia mid-diaphysis in hens from all treatment groups. Bone hydroxyapatite size was not affected by diet. Xylanase supplementation influenced the carbonate-to-phosphate ratio and crystallinity index in hens fed with both diets. Xylanase had more pronounced effects on bone mineral density and collagen maturity in hens fed with the rye–wheat–corn diet versus those fed with the wheat–corn diet. The results of this study showed that modern rye varieties, when supplemented with exogenous xylanase, can be introduced to the diet of laying hens without any adverse effects on bone structure.

## 1. Introduction

Bone tissue is composed of about 60% inorganic matter (calcium, phosphorus, and other minerals located mainly in the hydroxyapatite crystallite structures), 10% water, and 30% organic matter (ossein, which contains collagen fibers, proteoglycans, and other bone matrix noncollagenous proteins). Bone remodeling and maturation involves changes in both the inorganic and organic bone phases and is dependent on the interaction between bone cell activities (osteoblasts responsible for bone mineralization and osteoclasts responsible for bone resorption) and the intermolecular networks of collagen. Proper bone structural composition is crucial for ensuring its major functions, namely, providing mechanical support for locomotor functions, protecting vital organs, and maintaining mineral homeostasis [1]. In mature egg-laying hens, a specific type of bone, named medullary bone, appears in the endosteal surface of long bones. The medullary bone functions to store calcium phosphate and other minerals for eggshell production [2,3,4,5]. When the calcium stored in the mineral matrix of the medullary bone is moved to the oviduct to be used for eggshell calcification, the physical strength of the bones decreases [1,6]. Gregory and Wilkins [7] estimated that about 30% of commercial egg-laying hens suffer at least one bone fracture in their lifetime. Therefore, mineral-metabolism-related disorders and bone weakness in laying hens, which affect both the effectiveness of the production cycle and the overall welfare of the hens, have been serious problems for decades [8,9,10,11]. 

Over and above genetic, endocrine, and environmental (including the housing system) factors, nutrition is acknowledged to be the most important factor affecting the quality of bones in laying hens, with nutritional factors playing an important role in the regulation of bone homeostasis [12,13,14,15,16,17,18]. Among others, dietary energy source is widely connected to bone mineralization, remodeling, and mechanical strength [19,20]. Nowadays, corn is the most commonly used energy source in the diets of intensively reared laying hens. While modern varieties of cereal grains commonly cultivated in Europe, like wheat or rye, are good alternative energy sources that can be used as partial replacements for corn [21,22,23], the consumption of diets with a high-level incorporation of wheat or rye leads to an increase in the concentration of antinutritive factors (soluble nonstarch polysaccharides, NSPs), which cannot be utilized by monogastric animals [21,24,25]. NSPs increase intestinal digesta viscosity, which has unfavorable effects on gut transit time and gut motility, which in turn reduces the absorption of nutrients [26,27]. The major portion of NSPs in both wheat and rye are arabinoxylans, the antinutritional effects of which are attributed to both water-extractable arabinoxylans (their viscous properties in the gastrointestinal tract of birds) and water-unextractable arabinoxylans (their nutrient-encapsulating effects in cereal cell walls) [28]. While wheat can be introduced to the diet of laying hens without limits, the recommended rye content, due to its higher content of arabinoxylans, is limited to 10% [21,29]. The negative effects of NSPs can be partially overcome by the supplementation of exogenous NSP-degrading enzymes to poultry diets. These include endo-beta-1,4-xylanases, which degrade arabinoxylans. While the magnitude of this response varies from grain to grain, the addition of xylanase to wheat- and rye-based diets can reduce intestinal viscosity, improve nutrient uptake, and stimulate beneficial microbial growth [30,31,32]. Therefore, when using exogenous enzymes, the maximum content of rye in the diet of laying hens can be as high as 40% [29,33,34]. While some studies show that the inclusion of cereals to corn-based diets does not affect egg performance and feed conversion ratio (FCR) in laying hens [34,35,36], the presence of NSP-rich grains in poultry diets has been shown to affect bone quality parameters [19,37]. For bone homeostasis and remodeling, the reported malabsorption of calcium, as well as fat-soluble vitamins, like vitamin D, is particularly important [38,39].

Yet there are no studies that simultaneously compare the effects of the dietary inclusion of hybrid rye, the modern varieties of which are characterized by a reduced amount of antinutritive factors, specifically arabinoxylans [21,22,40], and xylanase supplementation on long bone quality in laying hens fed with a wheat–corn diet. In order to properly assess the effect of nutrition on bone properties, it is necessary to analyze the structure, organization, and characteristics of both the inorganic and organic components of the bone. 

Therefore, the current study was carried out to investigate the compositional changes in the tibia of laying hens fed with rye–wheat–corn and wheat–corn-based diets, with or without xylanase supplementation, in order to determine the interactive effects of rye and xylanase. Dual-energy X-ray absorptiometry (DXA), Fourier-transform infrared (FTIR) spectroscopy, and X-ray diffraction (XRD) techniques were used. These techniques allowed for the provision of detailed information concerning the mineralization, structural organization, and interaction of both the inorganic and organic bone phases of the tibia, which is considered a model bone in poultry research. Moreover, to visualize the distribution of collagen in the compact (structural) and medullary bones, Picrosirius red (PSR) staining was performed on decalcified, paraffin-embedded tissue sections of bone samples collected from the laying hens fed with the respective diets.

## 2. Materials and Methods

### 2.1. Ethical Approval

All laying hens were housed in the chicken facility of the Department of Animal Nutrition and Feed Science, National Research Institute of Animal Production, Balice, Poland. The experiment was carried out in compliance with the European Union law of the European Parliament and of the European Council (Directive 2010/63/UE, received in Poland by Legislative Decree 266/2015) on the protection of animals used for scientific purposes. According to the EU/PL law, approval from an animal ethics committee was not required, as the birds were fed a nontoxic diet and were not subjected to any invasive procedures. Polish regulations allow for this type of trial to be run on livestock animals without particular per case agreement by the local ethics committee, as long as no procedures are performed that might cause suffering, pain, or other stress, as was the case with the present study. All of the authors who took part directly in the planning and running of the experiment held certificates for the experimentation on living animals, delivered by the Polish Veterinary Services, as required by EU/PL law.

### 2.2. Birds and Experimental Diets

The experiment was carried out using 96 17-week-old commercial ISA Brown laying hens, obtained from a local hatchery. The hens were placed in wire-mesh floor cages (two hens per cage) and housed under climate-controlled conditions. Each cage provided 3600 cm^2^ of total floor space and was equipped with a claw-shortening device, two perches, one nipple drinker, and two feed troughs. During the pre-experimental period, which lasted from the 18th to the 25th week of age, the hens were fed a standard commercial diet. The experimental period took place from the 26th to the 50th week of age. The hens were randomly assigned to one of four dietary treatment groups, each comprising of 12 replicate cages, blocked in four columns and three rows. Feed, in a mash form, was offered ad libitum, and the hens also had free access to water and were exposed to a 14:10 (L:D) lighting schedule. 

A 2 × 2 factorial arrangement was employed, with rye inclusion (0% or 25%) and xylanase supplementation (0 or 200 mg/kg of feed) as factors. Hybrid winter rye cv. Brasetto and xylanase Ronozyme WX (DSM Nutritional Products Sp. z o.o., Mszczonów, Poland) with minimum xylanase activity, 1000 FXU/g, were used. The chemical composition of the rye grain included 9.23% crude protein, 0.81% crude fat, 1.46% crude fiber, and 1.51.% crude ash [41]. The experimental diets, isonitrogenous and isoenergetic, were formulated to meet or exceed the nutrient requirements of laying hens [29] (Table 1). The nutrient composition of the diets was calculated based on the chemical composition of the raw feedstuff, and the metabolizable energy value was calculated according to European tables [42], as the sum of the metabolizable energy (ME) content of the various components. The content of antinutritional factors in the experimental diets, as analyzed using gas chromatography, was as follows: wheat–corn diet: total NSPs—77.8 g/kg DM, insoluble NSPs—67.0 g/kg DM, arabinoxylans—33.4 g/kg DM; rye–wheat–corn diet: total NSPs—93.4 g/kg DM, insoluble NSPs—75.9 g/kg DM, arabinoxylans—40.84 g/kg DM [43].

At the end of the experiment period, one hen from each replicate cage was randomly selected and slaughtered by cervical dislocation, after electrical stunning. Immediately after slaughter, the right tibiae were carefully dissected out, cleaned of adhering tissues, and frozen in separate plastic bags at −20 °C until further examination.

### 2.3. Bone Measurements and Bone Sample Preparation

After thawing overnight at 7 °C, the tibiae were subjected to bone mineral density (BMD) and bone mineral content (BMC) measurements using dual-energy X-ray absorptiometry (DXA) on a Lunar iDXA densitometer (GE, Madison, WI, USA) located at the Lubelskie Centrum Diagnostyczne (Lublin Diagnostic Center), Świdnik, Poland. A high-resolution slow scan (scan mode: standard, 10.0 µGy) was used. Prior to the measurements, the apparatus was calibrated using bone phantoms of known BMD (BMD range of 0.6–1.4 g/cm^2^) designed for use on Lunar densitometers. A scan of the whole bone was performed using the “small animal” mode of the enCORE^®^ software (version 17.0 (2016); GE, Madison, WI, USA) and a special pad for “small animal” scans provided by the producer densitometer, which was used to eliminate measurement artifacts and to immobilize the bones so that all the bones were scanned in the coronal plane. No additional objects were used to mimic the soft tissue around bone [17,18,44]. The measurements were carried out on the scanned data using operator-defined regions of interest (ROIs) covering the central, 40 mm long fragment of the tibial bone, at the mid-diaphysis. Since these lines are fixed at specific intersections, a calculated compromise was consistently applied. All measurements (blind) were carried out by the same person.

Next, 5 mm thick transversally cut slices of bone diaphysis (containing both compact and medullary bones) were cut at the midpoint of the bone diaphysis, with an MBS 240/E diamond bandsaw (Proxxon GmbH, Foehren, Germany). The samples were then fixed in phosphate-buffered 4% formaldehyde (pH 7.0).

The other parts of the central fragment of bone diaphysis were cleaned for bone marrow in running water and then defatted using chloroform–methanol (2:1, *v:v*) at room temperature for 24 h under constant agitation (laboratory shaker Elpan 357, Elpin-plus, Lubawa, Poland). Next, a 10 mm long fragment was cut, dried for 24 h at a temperature of 105 °C and finally calcined in a muffle furnace at 500 °C for 24 h to determine the ash percentage, which was expressed relative to the sample dry weight. The remaining parts of the defatted bone sample were placed in a mortar, chilled in liquid nitrogen, and pulverized using a pestle until the bone was powdered. The powdered bone tissue was transferred to two 2.0 mL Eppendorf microtubes and frozen at −20 °C. The bone samples from the first microtube were designated to XRD measurements, from the second to FTIR spectroscopy.

### 2.4. X-Ray Diffraction (XRD) Measurements

The mean size of the hydroxyapatite and crystallinity of the raw bone were measured using X-ray diffraction (XRD). A high-resolution X-ray Empyrean diffractometer (PANalytical, Almelo, the Netherlands), with Cu K-alpha radiation (λ = 1.54178 Å) and a Ni filter at a generator voltage of 40 kV and current of 30 mA, was used to perform the structural characterization of the bone. The radiation was detected with a proportional detector. The samples were measured in θ–2θ geometry over a range from 10° to 80° with a step size of 0.01° and counting time of 6 s per data point. All measurements were carried out at room temperature. The source divergence and detector slit were 1/8, and Soller slits were applied. Bragg peaks and crystallographic planes were identified using a HighScore Plus software package (PANalytical, Almelo, the Netherlands) from the hydroxyapatite references (No. 96-901-0053). The sizes of the hydroxyapatite crystals’ crystallites and the degree of crystallinity were calculated using the Miller index (002). The peak position and the full width at half-maximum intensity (FWHM) were calculated from the fits of the Lorentz function using OriginPro 2016 software (OriginLab Co., Northampton, MA, USA) [45]. The calculated compositional parameters determined to evaluate bone material quality, derived from XRD spectroscopy, are characterized in Table 2.

### 2.5. FTIR Measurements

Infrared absorption spectra were recorded with a Vertex 70 FTIR (Bruker Optik, Ettlingen, Germany) spectrometer, in the attenuated total reflection (ATR) mode, equipped with a liquid nitrogen cooled MCT (mercury–cadmium–telluride) detector. A zinc selenide (ZnSe) crystal, with 20 internal reflections and an incidence angle of 45°, was the horizontal ATR element (PIKE Technologies). The spectrophotometer was equipped with a HeNe laser, which emits red light at 633 nm, with a power output of 0.8 mW. All ATR FTIR spectra were obtained at room temperature, in the spectral region, between 4000 and 900 cm^−1^. Typically, each spectrum represented an average of 64 scans taken at a resolution of 4 cm^−1^. The acquisition time necessary to generate an average spectrum of 64 scans was 30 s. The instrument was continuously purged with N_2_ for 40 min before and during measurements. The ZnSe crystal plate was cleaned with ultrapure organic solvents. Powdered raw bone samples were placed onto the face of the crystal of the ATR unit, and the tip of the clamp was pressed onto the crystal surface to make the necessary contact to obtain a spectrum. The spectra were analyzed using a commercially available software package, GRAMS/AI (Thermo Fisher Scientific, Waltham, MA, USA). After a baseline correction, smoothing by a 5-point Savitzky–Golay filter and normalization using the 960 cm^−1^ (the ν_1_ mode) band characterized by its intensity, overlapping peaks were resolved and their integrated areas measured using curve-fitting deconvolution with a Gaussian function [46]. The calculated compositional parameters determined to evaluate bone material quality derived from FTIR spectra analyses are characterized in Table 3.

**Table 2:** Parameters used in the XRD analysis of bone material quality of laying hens fed either a wheat–corn diet or rye–wheat–corn diet (25% of hybrid rye inclusion), nonsupplemented or supplemented with xylanase (200 mg/kg of feed) for a period of 25 weeks.

Parameter	Formula	Description	Reference
Size of the hydroxyapatite crystals in the c-axis	$D=\frac{K\lambda }{\beta cos\theta }$	K—a constant related to the crystallite shape (0.94), λ—the wavelength of X-ray radiation (1.54178 Å), β—the full width at half-maximum intensity (FWHM) of (002) reflection peak, counting the apparatus broadening of 0.08°, θ—the (002) peak position	[47,48]
Mineral crystallinity index	$CI={\left(\frac{K_{A}}{FWHM_{002}}\right)}^{3}$	K_A_—a constant set to 0.24, FWHM_002_—the full width at half-maximum intensity of (002) reflection peak	[49]

**Table 3:** Parameters used in the FTIR analysis of bone material quality of laying hens fed either a wheat–corn diet or rye–wheat–corn diet (25% of hybrid rye inclusion), nonsupplemented or supplemented with xylanase (200 mg/kg of feed), for a period of 25 weeks.

Parameter	Formula	Description	Reference
Mineral-to-matrix ratio	$M/M=\frac{\mathrm{Area}\left(v_{1},v_{2}PO_{4}^{3-}\right)}{\mathrm{Area}\left(\mathrm{Amide}I\right)}$	The ratio of the main phosphate (ν_1_, ν_3_, PO_4_^3−^; 900–1200 cm^−1^) to amide I (1600–1700 cm^−1^) integrated band area (Figure 1A).	[50]
Collagen maturity	$\mathrm{CM}=\frac{\mathrm{Area}\left(1660\;cm^{-1}\right)}{\mathrm{Area}\left(1690\;cm^{-1}\right)}$	The ratio of the area ratio between amide I sub-bands at mature cross-links (1660 cm^−1^) and immature cross-links (1690 cm^−1^) (Figure 1B).	[51,52]
Carbonate-to-phosphate ratio	$C/P=\frac{\mathrm{Area}\left(CO_{3}^{2-}\right)}{\mathrm{Area}\left({\mathrm{PO}}_{4}^{3-}\right)}$	The ratio of the integrated area of the carbonate spectral region (840–890 cm^−1^) to integrated area of the ν_1_, ν_3_, PO_4_^3−^ spectral region (900–1200 cm^−1^).	[46,52,53]
Crystallinity index	$\mathrm{CI}=\frac{\mathrm{Area}\left(1030\;cm^{-1}\right)}{\mathrm{Area}\left(1020\;cm^{-1}\right)}$	The ratio between phosphate sub-band areas of stoichiometric (at 1030 cm^−1^) to nonstoichiometric (1020 cm^−1^) hydroxyapatites (Figure 1C).	[54]

### 2.6. PSR Staining

Formaldehyde-fixed samples of tibial bone diaphysis were decalcified in a 10% EDTA solution (pH 7.4). Afterwards, the decalcified samples were dehydrated through a graded ethanol series (50%, 70%, 90%, and 100% ethanol in distilled water), fixed with nonpolar Ottix Plus and Ottix Sharper solvents (DiaPath, Martinengo, Italy) and embedded in paraffin. Paraffin-fixed samples were cut with an HM 325 microtome (Thermo Fisher Scientific, Waltham, MA, USA) at a thickness of 5 μm and stained with Picrosirius red (PSR) stain to observe the distribution of mature, organized (seen as red/orange), and immature (seen as green) collagen fibers [55]. Stained slides were observed in cross-polarized light using a CX43 (Olympus, Tokyo, Japan) microscope.

### 2.7. Statistical Analysis

The data were analyzed using a 2 × 2 factorial arrangement with rye inclusion and xylanase supplementation as the factors. The interaction between rye inclusion and xylanase supplementation was added to the model. A bird was considered an experimental unit. Normality of data was tested using the Shapiro–Wilk normality test, and homogeneity of variances was tested by Levene’s test. As all variables passed the normality test (*p*-value > 0.05), whenever significant differences were found among treatments, treatment means were analyzed using a Tukey’s HSD test. For all tests, a *p*-value < 0.05 and a confidence interval of 95% were established. The data were analyzed using Statistica software (v. 13.3, TIBCO Software Inc., Palo Alto, CA, USA).

## 3. Results

DXA analysis showed that the tibia from laying hens fed with the wheat–corn diet were characterized by a lower bone mid-diaphysis planar area compared with the tibia from hens fed the rye–wheat–corn diet (*p* < 0.001, Table 4). There was also a significant effect of xylanase, as the tibia from hens in xylanase-supplemented groups had a lower bone mid-diaphysis planar area than that from hens in enzyme-deprived groups (*p* < 0.001). For bone mineral density (BMD), there was an interaction between rye content and xylanase supplementation (*p* < 0.05). When xylanase was added to the rye–wheat–corn diet, a significant increase in BMD was observed. Moreover, hens fed the rye–wheat–corn diet without xylanase had significantly decreased tibial BMD compared with that of hens fed with the wheat–corn diet without xylanase (Table 4). As a result of the differences in BMD and bone planar area, there was a significant interaction between rye content and xylanase supplementation for bone mineral content (BMC) (*p* < 0.05). Hens fed with the wheat–corn diet had significantly decreased bone mineral content in the tibial bone diaphysis following xylanase supplementation (*p* < 0.05), what was not observed when hens were fed the rye–wheat–corn diet.

The bone quality data obtained from XRD and FTIR measurements are presented in Table 5. Raw bone XRD structural analysis of position and FWHM of the (002) reflection peak (Figure 2) showed that diet type had no effect on hydroxyapatite crystallite c-axis size or on bone crystallinity index (Table 5). On the other hand, significant effects of xylanase supplementation on bone compositional parameters were observed with regard to the FTIR spectra data (Table 5). In xylanase-supplemented groups, a reduction in crystallinity (*p* < 0.01) was observed; this effect was especially evident for the rye-fed group. There was also an effect of xylanase supplementation on carbonate-to-phosphate ratio, which was significantly decreased in xylanase-supplemented groups (*p* < 0.01). An interaction between diet type and enzyme addition for mineral-to-matrix ratio (main-phosphate-to-amide-I-area ratio) and collagen maturity was also noted. The mineral-to-matrix ratio, which is an indicator of the degree of bone mineralization, significantly increased when xylanase was introduced to the wheat–corn diet (*p* < 0.05). Collagen maturity (1660 and 1690 cm^−1^ sub-bans area ratio) was lowest in the bones from laying hens fed with the rye–wheat–corn diet with no xylanase and was significantly improved following xylanase supplementation to a value similar to those observed in both wheat–corn–diet fed groups.

**Figure 1:**
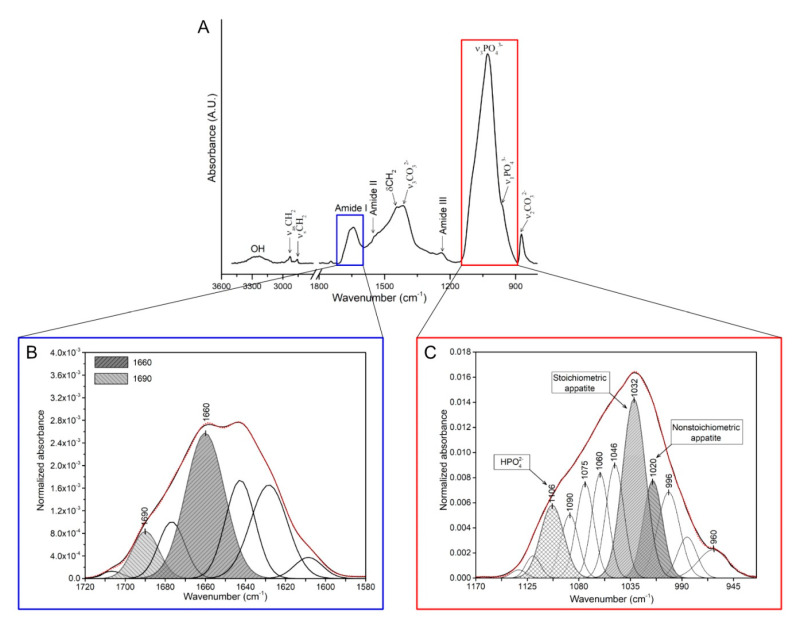
(**A**) Typical FTIR spectra of a raw bone sample showing the vibrational assignment of the most significant bands, and overlapping peaks constituting the phosphate group regions and amide I band. Protein secondary structure analysis. (**B**) Deconvolution of amide I region: baseline-corrected spectra were fitted with seven Gaussian band profiles by approximating the number and position, using the minima of second derivatives, which when simulated fits. The integrated area under sub-bands (1690 and 1660 cm^−1^) was used for the calculation of collagen maturity. (**C**) Deconvolution of the phosphate group regions: The ν_1_, ν_3_ PO_4_^3−^ band (stoichiometric apatite, nonstoichiometric apatite, respectively) with underlying component modes revealed by curve fitting. Integrated area under sub-bands (ν_1_, ν_3_ PO_4_^3−^, HPO_4_^2−^) were used for the calculation of crystallinity. The red dotted line simulates fit to the experimental curve, fitted with twelve Gaussian band profiles.

**Figure 2:**
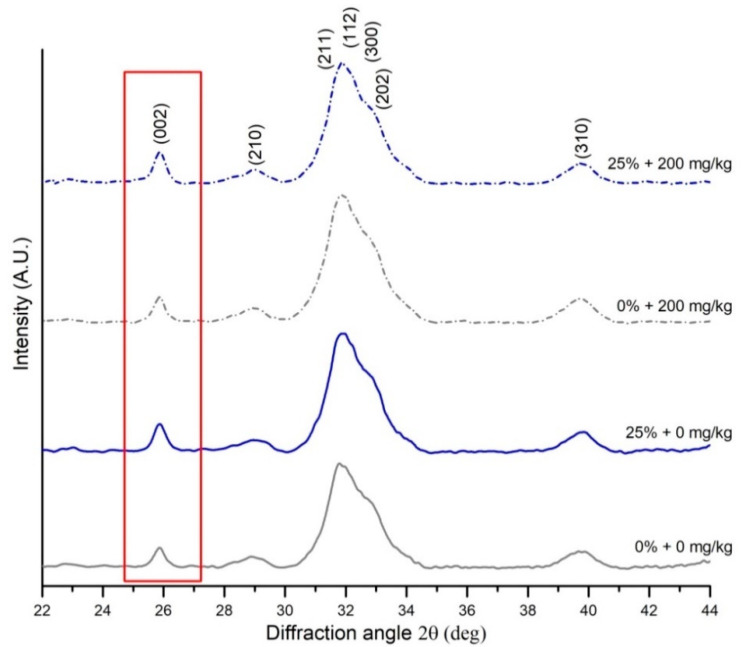
Representative XRD patterns of the raw bone samples from 50-week-old laying hens fed with either a wheat–corn diet or a rye–wheat–corn diet (25% of hybrid rye inclusion), nonsupplemented or supplemented with xylanase (200 mg/kg of feed), for a period of 25 weeks. The position and FWHM (full width at half-maximum) of the (002) reflection peak were used for the calculation of BAp (bioapatite) crystal size and crystallinity index.

**Figure 3:**
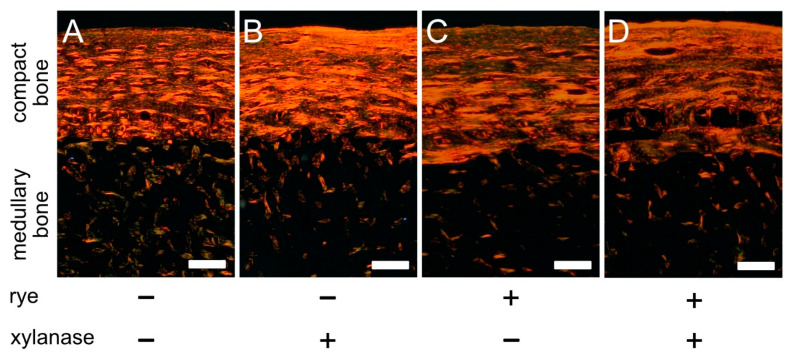
Representative photomicrographs of PSR-stained sections from the tibia mid-diaphysis of 50-week-old laying hens fed with either a wheat–corn diet (**A**,**B**) or a rye–wheat–corn diet (**C**,**D**) (25% of hybrid rye inclusion), nonsupplemented (**A**,**C**) or supplemented (**B**,**D**) with xylanase (200 mg/kg of feed), for a period of 25 weeks, observed in polarized light. Well-organized and more mature collagen fibers show orange-red birefringence, and the immature ones are green. A difference in the organization of collagen in compact bone in both xylanase-supplemented groups (**B**,**D**) characterized by a more ordered arrangement of mature collagen fibers can be observed. Also, in the medullary bone, both xylanase-deprived groups (**A**,**C**) show a less organized random arrangement of collagen fibers. All scale bars represent 100 µm.

Figure 3 shows representative images of Picrosirius red (PSR)-stained sections of the tibial bone mid-diaphysis, where both compact bone and medullary bone can be observed. Figure 3C shows an increase in the fraction of immature collagen fibers (green) in compact bone from hens fed with the rye-containing diet, without xylanase. Xylanase supplementation to the rye-containing diet resulted in a more mature, organized structure, similar to that observed in the wheat–corn–diet fed groups. Analogous changes, but to a lesser extent, were observed in the wheat–corn–diet group after the addition of xylanase (Figure 1A,B). In addition, a less organized, random arrangement of collagen was observed in the medullary bone of both groups deprived of xylanase supplementation (Figure 1A,C), compared with the groups that received xylanase (Figure 1B,D).

## 4. Discussion

In recent years, XRD and spectroscopic analyses have become more widely used as tools for analyzing the effects of environmental, endocrine, age-dependent, and nutritional factors on the organization of bone constitutive phases in different areas of veterinary and animal sciences [56,57,58,59,60,61,62]. The analysis of X-ray reflection peaks and spectroscopic bands allows us to assess both the structure and the nature of interaction of both the inorganic and organic bone matrixes, which include the assessment of, among others, the degree of bone mineralization, crystallinity, and collagen maturity. The present study made use of these techniques to evaluate the effects of dietary rye inclusion and xylanase enzyme supplementation on bone quality in egg-laying hens. The bones of laying hens are characterized by ongoing rapid bone remodeling processes, which affect bone function and architecture, as well as bone mineralization and metabolic activity. Potential negative changes in bone structural organization resulting from inappropriate nutrition may disturb bone remodeling processes, which could reduce the availability of minerals for eggshell calcification and, therefore, drastically influence eggshell and egg quality.

In our study, an increase in bone area (as measured using DXA) was observed after dietary rye inclusion. This may indicate that the circumference of the tibial mid-diaphysis had increased. However, one should remember that the DXA measurement gives the area of the planar projection of the bone. The cross sections of the tibia mid-diaphysis in poultry is roughly elliptical [63]. Therefore, an increase in diameter in one plane does not necessarily indicate an increase in diameter in the other plane as well. While there are no similar studies on laying hens, studies using broiler chickens and turkey poults fed with rye-based diets (37% and 58% of rye, respectively) showed decreased tibial diameter in birds fed with rye diet when compared with birds fed with corn diet [64,65]. A previous study by our lab on broiler chickens showed that dietary rye inclusion (15%) had no effect on bone quality (in terms of BMD and bone cross-sectional area), and the addition of 200 mg/kg xylanase (1000 FXU/g) increased the tibial BMD in chickens fed with both diets [66]. The results of the present study indicate that the same dose of xylanase, when supplemented to the 25% rye–wheat–corn-based diet, also increased the tibial BMD of laying hens.

We also aimed to examine whether dietary rye inclusion or xylanase addition could alter bone the hydroxyapatite structure in laying hens, as well as the crystallinity or hydroxyapatite crystal domain size influence on bone properties [67,68]. There are some recent studies showing that diet type influences bone crystal size in pigs [56,57]. There is also a previous study on laying hens where crystallinity was analyzed; however, in this particular study, age-related changes were examined [59]. In the current study, FTIR spectroscopy showed that xylanase addition reduced the crystallinity of the bone mineral phase of the tibia in laying hens fed with a wheat–corn diet. Generally, bone crystallinity, as measured by FTIR, can be confirmed by XRD analyses based on the FWHM of the (002) reflection peak [69]. This, however, was not observed in our study as XRD diffraction showed no differences in crystallinity index between groups. The differences in crystallinity determined with FTIR and that determined by XRD methods are probably due to fact that the measurements were performed on raw bone samples, and the XRD measurement of FWHM is dependent on the presence of organic matter in the bone matrix [48] as the (002) reflection peak also represents a measurement of the alignment of mineralized collagen fibrils [70]. Finally, while in mammals a correlation between the degree of bone hydroxyapatite crystallinity and bone mineral density is generally reported [71,72], this relationship was not observed in the current study. This is because in mammals and male birds, the measurement of the femoral mid-diaphysis bone density involves the measurement of the bone mineral density of the structural cortical bone only, while in egg-laying hens, the BMD measurement of the same region of the tibia gives the sum of the mineral densities of both the cortical and medullary bones [73], which significantly differ in bone turnover rate, and in general, the medullary bone has low BMD for rapid osteoclastic bone resorption.

An important observation from the XRD data is that there was no effect of dietary treatment on hydroxyapatite crystal sizes. No changes in crystal size suggests that all the bones assessed show the same balance in the management of eggshell-production-related high requirements of Ca and in bone remodeling rate, as suggested by Li et al. [59]. Moreover, it is well documented that osteoporotic bone displays larger crystallite size of the hydroxyapatite mineral, which can result in more fragile bones, with reduced stiffness [74]. Therefore, the lack of differences in hydroxyapatite crystal size between groups in our study indicates that the dietary treatments should have no effect on bone elasticity. This aspect of the XRD data should be further examined. For example, hydroxyapatite crystallite c-axis size can be validated using data collected on the (004) reflection peak [75]. However, to date, these data have generally been used for analyses concerning synthetic hydroxyapatites [76] or the hydroxyapatite present in tooth enamel [75]. Crystallinity data are also dependent on the presence of impurities in bone hydroxyapatite. Li et al. [59] observed a rapid increase in the incorporation of carbonate into the cortical bone of laying hens during the laying period. As shown by FTIR analyses, our hens, at the age of 50 weeks, displayed different tibial-carbonate-to-phosphate ratios between groups, which could be a potential trigger for osteoporosis [59,77]. Finally, according to the literature, bone-mineral-to-matrix ratio is related to the mineral content expressed in terms of bone ash percentage [69]. The same was observed in our study (Table 4 and Table 5), where xylanase supplementation had a positive effect on hens fed with the wheat–corn diet, and the dietary rye inclusion had no negative effect on the bone-mineral-to-matrix ratio. However, this was not observed for BMD determined using the DXA method. This may be due to the limitations of DXA as a method for determining bone mineral content, which include not only the abovementioned fact that DXA estimates the content on the basis of 2D scans of bone but also the fact that it has been shown that the result of DXA may depend on whether the scan is made for a whole limb or an isolated bone. However, while both types of scan in laying hens are highly correlated [78], Baird et al. [16] found that DXA underestimated the BMC of excised tibias from 58-week-old hens comparing bone ash; the opposite was observed by Schreiwes et al. [79], where DXA measurement of intact limb overestimated the BMC in tibias of 58-week-old live hens. To sum up the results of the XRD and FTIR measurements, no significant changes in the structural organization of the bone mineral phase was observed in 50-week-old laying hens, irrespective of dietary treatment, while FTIR spectroscopy showed that hens fed with the rye–wheat–corn diet showed the same structural organization of bone constitutive phases, irrespective of xylanase supplementation.

Both the amount of mineralized bone and the collagen cross-linking contribute to the quality of bone in laying hens [80], with bone loss being associated with significant changes in the organization of the cortical (structural) bone collagenous matrix [69]. Bone mechanical strength in birds was found to be correlated with the level of immature cross-links in the cortical bone [81]. Compared with other long bones in the avian skeleton, the long bones of the pelvic limb are characterized by a significant proportion of immature collagen, which reflects their physiological function as a calcium deposit [69]. However, as shown in PSR-stained sections (Figure 3), the cortical bone of hens supplemented with xylanase in the current study showed a significant reduction in the area of green, immature cross-links associated with newly synthesized collagen, indicating a higher bone turnover rate [69]. This shows that xylanase supplementation to the rye-based diet affects the collagen formation process, thereby leading to the formation of more a mature bone. In a healthy mature bone, an increase in collagen cross-linking has been correlated with an improvement in bone strength and increased resistance to fracture in laying hens [69]. This may also indicate that xylanase supplementation increased bone strength irrespective of dietary rye inclusion, as has previously been observed for broiler chickens fed with wheat–corn and rye–wheat–corn diets [66]. On the other hand, Silversides et al. [32] showed that supplementation of xylanase (2000 U/kg) to a wheat–corn diet did not affect bone breaking strength in laying hens at 64 weeks of age. Thus, bone strength should be further investigated in future studies.

In the medullary bone, a reduction in green-stained collagen cross-links was observed after xylanase supplementation (Figure 3). The medullary bone has a very high rate of formation and resorption [3]. Adequate regulation of mineral resorption in the medullary bone is essential for calcification of the eggshell [2]. Yet without detailed analysis of the medullary bone, we are unable to conclude as to how this reduction in immature collagen influenced the eggshell formation process. However, while the decrease of the egg-laying rate in rye–wheat–corn groups was observed (95.6% and 95.2% in xylanase-deprived and xylanase-supplemented groups, respectively) when compared with wheat–corn groups (97.2% in both groups), both rye content and xylanase addition had no effect on eggshell weight and density of the eggs laid by our hens, as has previously been reported [43]. On the other hand, we observed an increase in the presence of more organized, mature cross-links, suggesting a reduction in the amount of labile carbonate and an increase in crystalline apatite crystals in the medullary bone [70]. Since there is a correlation between mineral crystallinity, collagen maturity, and mineral content [73], the increase in collagen maturity observed in both the cortical and medullary bones in xylanase-supplemented groups is probably responsible for the observed increase in BMC in the hens fed with wheat–corn diet after xylanase supplementation and the increase in BMD in the hens fed with rye–wheat–corn diet, despite the observed reduction in bone cross-sectional planar area.

As shown by PSR staining, dietary treatment influenced collagen cross-links in the medullary bone. The medullary bone is highly reactive and more metabolically active and thus has a much higher turnover rate than the compact bone. Due to the high turnover rate, there are always areas of the medullary bone that differ with regard to mineralization and hydroxyapatite crystals composition [50]. Our results show that future XRD and FTIR studies that are conducted separately for compact and medullary bone tissues are needed. However, our study provides an important first step in the characterization of the previously unknown relationship between diet type and structural organization of bone constitutive phases in laying hens and can guide future investigations with regard to the function of enzyme supplementation on bone quality in poultry. Nevertheless, in this study, XRD and FTIR spectroscopy revealed their power in investigating structural changes in bone constitutive phases in the bones of laying hens as a result of dietary treatments, showing that in combination with histological examination, these techniques can provide important information concerning bone mineral and organic matrix composition, crystallinity, and collagen maturity.

## 5. Conclusions

To the best of our knowledge, this is the first study that shows that the structural organization of the bone can be affected by dietary factors in laying hens. We found that xylanase supplementation to both grain diets resulted in improved bone structure. The nature of the response to xylanase is dependent on the type of grain, but regardless of diet type, the response is positive. As an effective food additive, xylanase can probably promote the absorption of minerals to enhance calcium homeostasis, which enhances bone quality. Finally, our study shows that modern rye verities can be introduced to the diet of laying hens; however, xylanase enzyme supplementation is recommended. However, to fully assess the effect of rye inclusion on calcium homeostasis in laying hens, further studies focusing on a detailed analysis of bone geometry, mineral composition, mechanical endurance, and eggshell quality need to be carried out.

## Figures and Tables

**Table 1:** Composition and calculated nutritional value of experimental diets fed to laying hens for a period of 25 weeks.

Item	Diet	
	Wheat–Corn	Rye–Wheat–Corn
*Ingredients (g/kg)*		
Rye	0.00	250.00
Wheat	382.30	250.80
Corn	290.00	150.00
Soybean meal	200.00	210.00
Rapeseed oil	15.00	27.00
Limestone	91.00	90.00
Monocalcium phosphate	12.00	12.00
NaCl	3.00	3.00
DL-Methionine	1.10	1.10
L-Lysine hydrochloride	0.60	0.10
Vitamin–mineral premix ^1^	5.00	5.00
Metabolizable energy, MJ/kg ^3^	11.55	
*Nutrient composition (g/kg DM) ^2^*		
Crude protein	170.00	
Lys	7.20	
Met	3.40	
Ca	36.00	
Available P	3.75	
Na	1.50	
Cl	2.19	

^1^ The premix provided per 1 kg of diet: vitamin A, 10,000; vitamin D3, 2000 IU; vitamin E, 20 IU; vitamin K3, 1.5 mg; vitamin B1, 1.0 mg; vitamin B2, 4.0 mg; vitamin B6, 1.5 mg; vitamin B12, 0.01 mg; Ca pantothenate, 8.7 mg; niacin, 20.0 mg; folic acid, 0.8 mg; choline chloride, 200.0 mg; manganese, 85.0 mg; zinc, 60.0 mg; iron, 45.0 mg; copper, 8.0 mg; iodine, 1.0 mg; selenium, 0.25 mg; ^2^ calculated according to the chemical composition of feed components; ^3^ calculated according to European tables [42] as the sum of the ME content of the various components.

**Table 4:** DXA measurements and tibia mid-diaphysis ash content of 50-week-old laying hens fed with either a wheat–corn diet or rye–wheat–corn diet (25% of hybrid rye inclusion), nonsupplemented or supplemented with xylanase (200 mg/kg of feed), for a period of 25 weeks.

Factors ^1^		DXA Measurement			Ash
Rye	Xylanase	BMD, g/cm^2^	Area, cm^2^	BMC, g	Ash, %
*Treatment ^2^*					
0%	0 mg/kg	0.275 ^b^	4.14	1.151 ^b^	29.9 ^a^
	200 mg/kg	0.238 ^ab^	3.62	0.902 ^a^	34.2 ^b^
25%	0 mg/kg	0.198 ^a^	4.57	0.899 ^a^	33.0 ^b^
	200 mg/kg	0.246 ^b^	4.12	1.012 ^ab^	31.7 ^ab^
*SEM ^3^*		0.018	0.09	0.081	0.6
*Main factors*					
0%		0.257	3.88 ^a^	1.027	32.4
25%		0.222	4.33 ^b^	0.956	32.1
	0 mg/kg	0.236	4.34 ^a^	1.025	31.5
	200 mg/kg	0.243	3.87 ^b^	0.957	33.0
*p*-value					
Rye		0.067	<0.001	0.392	0.603
Xylanase		0.722	<0.001	0.409	0.020
Rye x xylanase		0.027	0.592	0.034	<0.001

^1^ Number of observations included in the calculation of means for treatment effects is 8, whereas means for main factors are 16. ^2^ Columns with different superscripts (^a, b^) are significantly different (*p* < 0.05). ^3^ SEM = standard error of the means, BMD = bone mineral density, BMC = bone mineral content.

**Table 5:** Structural organization of bone constitutive phases of the tibia mid-diaphysis of 50-week-old laying hens fed with either a wheat–corn diet or a rye–wheat–corn diet (25% of hybrid rye inclusion), nonsupplemented or supplemented with xylanase (200 mg/kg of feed), for a period of 25 weeks.

Factors ^1^		XRD Diffraction		FTIR Spectroscopy			
Rye	Xylanase	HA c-Axis Size, nm	Crystallinity Index	Mineral-to-Matrix Ratio	Carbonate-to-Phosphate Ratio	Crystallinity	Collagen Maturity
*Treatment ^2^*							
0%	0 mg/kg	19.28	0.161	10.57 ^a^	0.0263	1.19	5.25 ^b^
	200 mg/kg	19.44	0.165	12.85 ^b^	0.0239	1.15	5.31 ^b^
25%	0 mg/kg	19.47	0.165	12.75 ^b^	0.0276	1.25	4.06 ^a^
	200 mg/kg	19.72	0.172	11.62 ^ab^	0.0250	1.17	5.36 ^b^
*SEM ^3^*		0.28	0.070	0.55	0.0007	0.03	0.18
*Main factors*							
0%		19.36	0.163	11.71	0.0251	1.17	5.28
25%		19.60	0.169	12.19	0.0263	1.21	4.71
	0 mg/kg	19.37	0.163	11.66	0.0270 ^b^	1.22 ^b^	4.65
	200 mg/kg	19.58	0.168	12.23	0.0245 ^a^	1.16 ^a^	5.34
*p*-value							
Rye		0.406	0.398	0.396	0.118	0.114	0.005
Xylanase		0.466	0.465	0.310	0.002	0.033	0.001
Rye x xylanase		0.871	0.842	0.005	0.876	0.523	0.002

^1^ Number of observations included in the calculation of means for treatment effects is 8, whereas means for main factors are 16. ^2^ Columns with different superscripts (^a, b^) are significantly different (*p* < 0.05). ^3^ SEM = standard error of the means. HA = bone hydroxyapatite.
